# Antibiotic use in acute mesenteric ischemia: a review of the evidence and call to action

**DOI:** 10.1186/s12959-023-00486-3

**Published:** 2023-04-11

**Authors:** Yuqian Tian, Sanjeev Dhara, Christopher D. Barrett, Aaron P. Richman, Tejal S. Brahmbhatt

**Affiliations:** 1 Division of Acute Care Surgery and Surgical Critical Care, Department of Surgery, University of Nebraska Medical Center, Omaha, NE USA; 2 Division of Vascular Surgery, Department of Surgery, Emory University, Atlanta, GA USA; 3 Divisions of Trauma and Acute Care Surgery and Surgical Critical Care, Department of Surgery, Boston University Medical Center, Boston University School of Medicine, Boston, MA USA

**Keywords:** Mesenteric ischemia, Thromboembolism, Antibiotics, Epithelial barrier, Sepsis

## Abstract

Acute mesenteric ischemia (AMI) is a life-threatening condition with a high mortality rate. The standard practice after making the diagnosis includes aggressive resuscitation, anticoagulation, followed by revascularization and resection of necrotic bowel. The role of empiric antibiotics in the management of AMI is not well defined in the literature. This review article aims to examine our current understanding on this matter, based on bench research and clinical studies. It is demonstrated in animal study model that the ischemia/reperfusion (I/R) injury damages intestinal epithelium, and subsequently lead to barrier dysfunction, a condition that can support bacterial translocation through a complex interplay between the intestinal epithelium, the intestinal immune system and the intestine’s endogenous bacterial population. Based on this mechanism, it is possible that the use of antibiotics may help mitigate the consequences of I/R injury, which is examined in few animal studies. In clinical practice, many guidelines support the use of prophylactic antibiotics, based on a meta-analysis of randomized control trials (RCTs) demonstrating the benefit of antibiotics in multi-organ dysfunction syndrome. However, there is no direct reference to AMI in this meta-analysis. Most clinical studies that focus on AMI and mentions the use of antibiotics are retrospective and single institution, and very few comments on the role of antibiotics in their discussions. We conclude that there is limited evidence in literature to support the use of prophylactic antibiotic in AMI to improve outcome. More clinical studies with high level of evidence and basic science research are needed to improve our understanding on this topic and ultimately help build a better clinical pathway for patients with AMI.

## Background

AMI is a life-threatening condition with reported mortality rates greater than 50% [1]. Even for patients who manage to survive the index hospitalization, there is a high readmission rate with a reported mortality rate of ~ 7% [2]. AMI can be subdivided by precipitating etiologies: mesenteric arterial embolism (40 to 50% of cases), mesenteric arterial thrombosis (20 to 35% of cases, frequently “acute-on-chronic”), mesenteric venous thrombosis (5 to 15% of cases), and non-occlusive mesenteric ischemia (5 to 15% of cases) (NOMI) [3]. A prior meta-analysis by Schoots et al. showed that the post-surgical mortality rate for AMI due to mesenteric arterial thrombosis was roughly 20% higher than for AMI due to mesenteric arterial embolism [4]. Typical presenting symptoms include pain out of proportion to exam, however the condition’s high mortality rate is partially driven by its relatively non-specific presentation, with a broad differential diagnosis encompassing most causes of an acute abdomen. This non-specific presentation and accompanying delays in diagnosis comes at a serious cost, as there is a significantly decreased mortality (10–20%) if AMI is diagnosed within 6 h of presentation [5]. At present the best diagnostic test is a computed tomographic angiography (CTA) due to its relatively rapid and noninvasive nature as well as high accuracy [6]. To date there are no specific, clinically validated serum biomarkers for the condition, although serum lactate and laboratory markers of acidosis can corroborate suspicion, and D-dimer has been shown to be useful as an exclusion test but lacks specificity [7–10].

Despite AMI’s high mortality, there is a relative paucity of high-level evidence to guide clinical practice [1, 11, 12]. In general, existing clinical practice guidelines emphasize obtaining a CTA rapidly, aggressive fluid resuscitation, correction of electrolyte abnormalities, and therapeutic anticoagulation. Therapeutic anticoagulation is particularly indicated for AMI caused by mesenteric venous thrombosis as the first line treatment. Interventions to restore venous flow is not usually required for this subgroup. For both mesenteric arterial embolism and thrombosis, the standard practice is to proceed with surgical interventions to restore blood flow emergently, but in clinical practice, therapeutic anticoagulation will be initiated as a bridging therapy immediately after diagnosis being established or when there is high suspicion clinically, to prevent worsening clot burden while awaiting final surgical plan. The first choice of therapeutic anticoagulation is usually unfractionated heparin, in anticipation of emergent operation and safe for patients with AKI which is not uncommon in AMI presentation. Therapeutic anticoagulation is not necessarily indicated for NOMI, since the etiology for this subgroup is not clot burden [1, 12]. Modern standard practice trends reflect that patients with generalized peritonitis should be taken for emergent laparotomy, while patients without peritonitis should be prioritized for urgent re-vascularization procedures and consideration for laparotomy to visually assess bowel viability. There have been more recent reports of endovascular approaches as the preferred method for managing AMI of arterial thrombotic or embolic origin, however this remains an open area of discussion regarding best practice [1, 3, 13, 14]. The proliferation of endovascular approaches has also led to renewed discussion regarding the timing of endovascular intervention and laparotomy for bowel viability in the patients without generalized peritonitis with multiple studies endorsing endovascular revascularization prior to laparotomy for visual inspection of bowel viability, otherwise known as an “endovascular first” approach [15–17]. This data however remains largely retrospective, limiting the evaluation of an endovascular first approach on patient outcomes.

While the procedural and surgical management of AMI is largely based on decades of surgical experience with the addition of newer endovascular options, little debate exists with respect to the fundamental tenet that vascular patency and flow must be restored, and non-viable bowel be resected. However, a significant knowledge gap still exists regarding the role of empiric antibiotics, where there is a lack of high-level evidence by way of clinical trials to support or refute their role in management of AMI, particularly when viscera are threatened but not necessarily non-viable. Multiple recently written guidelines do endorse the universal usage of broad spectrum antibiotics in AMI, although they too note that there is absence of data to guide this [1, 5, 12, 18, 19]. This relative lack of detailed examination regarding antibiotic usage may relate to the high proportion of cases in which AMI presents with conditions such as sepsis or septic shock, at which point other standardized clinical decision-making regimens dictate antibiotic usage. However, in one study roughly 66% of patients with AMI did not end up requiring a bowel resection on the initial exploratory [20]. Furthermore, it is likely that a significant fraction of patients who do undergo resection for AMI do so because non-viability is questioned rather than confirmed. In both these cases, antibiotics may have a particularly important, yet unproven, role to play. Further complicating the case for antibiotics in AMI are the risks that antibiotics carry such as increased rate of *C. diff*, renal and hepato-toxicities, as well as the general importance of antibiotic stewardship in critically ill [21–23]. The decision regarding antibiotic use is highly variable between clinicians despite the clinical problem being routinely present on most general/acute care and vascular surgical services. Management of patients without sepsis or generalized peritonitis with AMI where there is a lack of frankly necrotic/inviable bowel presents a dilemma for antibiotic indication. The evolution in the management of appendicitis is a useful corollary that suggests how the treatment of AMI might evolve with further studies specifically examining the role of antibiotics. Historically the management of acute uncomplicated appendicitis has been an appendectomy however a raft of RCTs and case series suggested the non-inferiority of an antibiotics-first approach for the condition similar to that of acute uncomplicated [24]. These studies were buttressed by a larger multicenter study from the Comparison of Outcomes on Antibiotic Drugs and Appendectomy (CODA) collaborative that again demonstrated the non-inferiority of antibiotics for uncomplicated appendicitis. The CODA study showed that of the antibiotic-only patients, only 3 in 10 required subsequent appendectomy within 90 days and roughly half did not require hospitalization on their initial presentation, thus significantly reducing the morbidity associated with [25].

Accordingly, this review examines the scientific and clinical literature regarding the role of antibiotics in AMI and ischemia/reperfusion (I/R) injury to the intestines. Furthermore, in absence of level I evidence, this review should also serve as a call to action for the community of intensivists, vascular surgeons, and acute care surgeons to further examine this question with high quality, prospective trials given that antibiotic therapy is not without risk.

## AMI and sepsis in the scientific literature

**Fig. 1:**
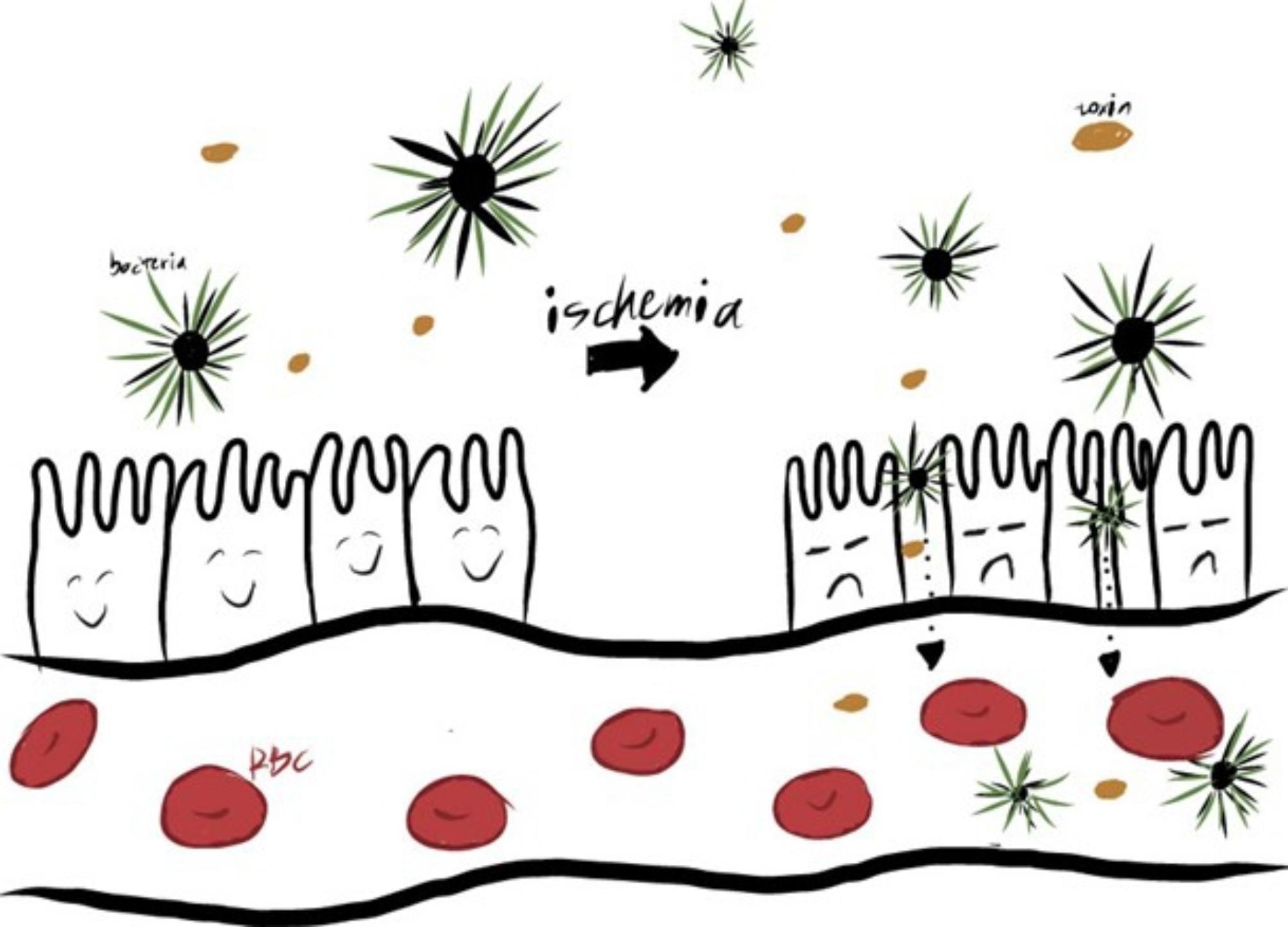
Visual illustrate of a “leaky gut” for bacterial translocation during I/R injury

A growing body of scientific literature posits that the gut is central to the pathophysiology of critical illness, and can be thought of as a “motor”[26]. This relationship is evident when examining the high rates of sepsis associated with AMI as compared to other arterial occlusion syndromes, such as myocardial infarction or stroke, which suggests a “specific septic component” to [27]. One of the earliest recorded links between critical illness and the gut noted the presence of bacterial endotoxin in patients in hemorrhagic shock. The authors went on to hypothesize that during periods of shock the body is uniquely vulnerable to the translocation of gram negative bacterial [28]. These early reports gave rise to the theory of a “leaky gut”, that when damaged allows for bacterial translocation and systemic infection, as illustrated in Fig. 1. This theory was bolstered with reports such as a canine study, in which survival rates with the superior mesenteric artery clamped for 7 h were increased in the group of canines given [29]. A similar study conducted in rats undergoing hemorrhagic shock lent further credence to the theory that “shock physically perturbs the normal barrier function of the mucosa” thus explaining how “shock results in bacterial translocation and endotoxemia”[30]. Despite evidence, there remained continued skepticism, as documented by a clinical study in which sequential portal vein sampling from 20 trauma patients did not show evidence of portal or systemic bacteremia despite a relatively high rate of subsequent multiple organ [31].

A more recent body of literature suggests an interplay between the intestinal epithelia, microbiota, and mucosal immune cells can explain the pathophysiological impact of ischemia/reperfusion injury. An early study examined the impact of I/R injury on the barrier function of rat intestinal and endothelial tissue through measurement of the flow or radiolabeled albumin as well as visualization of tissue through electron microscopy. It found that the period of ischemia with subsequent reperfusion correlated with the degree of epithelial barrier (and endothelial) [32]. Further study regarding the mechanistic link between barrier dysfunction and bacterial translocation has shown that intestinal inflammation can trigger transcellular migration of normally non-invasive bacterial species even prior to the disruption of intestinal tight junctions, which had long been purported as the avenue through which bacteria move past the epithelial [33]. Beyond inducing barrier dysfunction, I/R injury has also been shown to markedly impact the numbers as well as phenotypes of murine gut-associated lymphoid [34]. As the impact of the microbiome has gained further attention, an increasing awareness has developed that the microbiome itself impacts and interacts with the various tissues, especially the intestinal ones that it resides next to. This can be attested by recent literature that shows that the response of murine neutrophils to I/R injury is different based on host colonization [35]. This notion is also supported by a recent study that demonstrated a protective effect of dexmedetomidine in alleviating intestinal I/R injury by modulating the gut flora [36, 37]. The aforementioned studies taken in sum constitute powerful evidence that the response of the gut to I/R injury can be explained by the “complex crosstalk” between the “the intestinal epithelium, the intestinal immune system and the intestine’s endogenous bacteria”[26].

Experiments in animals have also offered insights in the potential role that antibiotics may play in mitigating the damages associated with I/R injury in AMI. A significant proportion of the injury in I/R has been shown to relate to the reperfusion of ischemic tissue, during which time reactive oxygen species and other harmful inflammatory cytokines further compound ischemic damage. A trial examining the impact of intestinal microbiota in murine response to I/R injury revealed that while conventional mice had marked intestinal and pulmonary inflammatory responses with “100% lethality,” germ-free mice did not show marked inflammation and experienced no lethality with the same [38]. Somewhat disappointingly, this same trial showed that conventional mice treated with antibiotics whose stool was bacteria negative still had a similar inflammatory response and lethality to untreated conventional [38]. Contrary to the previous study, a subsequent murine study in which antibiotic treatment preceded I/R injury showed that antibiotic treatment “attenuated intestinal [I/R] injury” with the authors encouraging efforts that involve “manipulation of the gut flora with probiotics [or] antibiotics” in patients who suffered I/R [39]. From a biological standpoint, even when intestinal viability remains, the epithelial barrier function is nonetheless compromised by I/R injury and leads to direct bacterial exposure of the lamina propria and submucosa, where much of the intestinal innate immunocytes reside (e.g. macrophages, dendritic cells, lymphocytes) and blood vessels to deliver acute inflammatory cells like neutrophils travel through. Such direct exposure of bacteria to a normally sterile tissue space occupied by immune cells and inflammatory cell conduits inevitably leads to significant inflammation as the body works to prevent bacteremia and sepsis, a response that is both necessary but also potentially damaging to the host tissues, a pyrrhic victory of sorts. It is plausible then, although unproven, that antibiotic therapy would aid in preventing both an overwhelming inflammatory response as well as bacteremia. In clinical conditions like ischemic colitis, where repeated episodes are known to cause significant fibrosis/scarring of the effected colon segments which itself begets further ischemic susceptibility, early antibiotic initiation may prevent the latter phenomena although this, too, is [40].

## Review of the clinical literature

Despite the previously discussed purported biological mechanism between gut ischemia and increased risk for mucosal translation along with studies suggesting a role for antibiotics in the treatment of AMI, to date there have been very few studies examining the role of prophylactic antibiotics. We summarize some of the more recent literature regarding AMI with a focus on antibiotics in Table 1. It is notable that much of the literature is composed of retrospective single institution studies. As outlined in Table 1, many of the studies do not mention antibiotics at all, while few studies had one sentence mentioning antibiotics use in method, but no further discussion related to antibiotics in the rest of texts. Examining some of the recent literature that is summarized in Table 1, two additional trends seem evident. In recent years, there have been multiple trials that document the proliferation of intestinal stroke centers, seemingly mirroring clinical care pathways that already exist for neurological stroke or myocardial [41–44]. These studies endorse this development and believe intestinal stroke centers can improve AMI outcomes. In addition, much of the most recent literature is meant to provide more information regarding the role of endovascular therapy and its relative efficacy versus open surgery.

As mentioned previously, multiple clinical practice guidelines do endorse antibiotic usage in [1, 5, 12, 19]. Interestingly, many cite a 2010 systematic meta-analysis of RCTs investigating oropharyngeal and intestinal administration of antibiotics and their impact on multi organ dysfunction [45]. The trials included were examining the use of antibiotics in populations ranging from transplant to pediatric surgery, however they did not include AMI. The meta-analysis focused on multiple organ failure and purported that the antibiotic regimens in the included RCTs were likely effective because they stop “infection, gut overgrowth, [gram negative bacteria] translocation, and endotoxin absorption”[45]. As such, despite not explicitly focusing on AMI, the meta-analysis advances the same thesis that antibiotics are helpful to prevent bacterial translocation and endotoxin release that can result in septic shock. It is important to note that the full protocol of selective digestive decontamination, followed by the RCTs in the study, includes both parenteral (“to control primary endogenous infections”) and enteral (“to control secondary endogenous infections of lower airways and blood”) antibiotic administration.

Of the studies explicitly mentioning antibiotic usage in AMI, a few are worth discussing more extensively. Corcos et al. (2013) described the workflow that they used to build an intestinal stroke center that incorporated evidence that oral digestive decontamination prevents multi-organ dysfunction [41, 45]. Their medical protocol involves oral digestive decontamination with oral gentamicin and metronidazole for all patients suspected of having AMI. In addition, for patients with systemic inflammatory response syndrome (SIRS) or organ failure they administered IV piperacillin-tazobactam. They followed their management protocol in 18 patients, and note that 11 of the patients did not ultimately require intestinal resection, a very high proportion, and postulate that the early initiation of a medical protocol that “[ed]…bacterial or inflammatory [s]” potentially reduced the need for [41]. Building upon this work and recognizing that prevention of irreversible transmural intestinal necrosis is the treatment goal in AMI, Nuzzo et al. aimed to investigate factors that would delay deleterious progression of [27, 41]. They retrospectively analyzed patient data from the same intestinal stroke center as Corcos et al. (2013) [27, 41]. They find “a significant protective effect” for oral antibiotics against irreversible transmural intestinal necrosis and suggest that systematic use of antibiotics motivated by the desire for oral digestive decontamination should be placed alongside anticoagulation and arterial revascularization as mainstays of [27]. In addition to these two more relevant studies, two other studies briefly mentioned antibiotics use in the result session, but no discussion was further developed. Caluwaerts et al. [46] included antibiotics use in one of the result tables as a possible prognostic factor of AMI in ICU patients. Based on retrospective data, however, it showed the use of antibiotics was equal in both people who survived and people who died from AMI. On the contrary, Jagielski et al. [11] found that antibiotics use was more commonly seen in the group of patients who eventually died from AMI. But there was no comment regarding the selection criteria used to decide who to receive antibiotics use. It is hard to decipher the clinical significance of the results of antibiotic use from these studies due to lack of information.

Examination of Table 1 suggests that more work needs to be done to specifically examine the role of antibiotics in AMI particularly in the form of RCTs and other high-level evidence.

## Conclusion and a call to action

The corollary from antibiotics evolution in management of acute appendicitis as well as the sparse mention of antibiotics in the AMI literature suggests a need for more high-level evidence examining antibiotics role in AMI. Even though already incorporated in many pathways or guidelines in managing AMI, the clinical benefit of antibiotics use has yet to be proven. Based on our discussion above, both basic science studies and clinical studies are critical for building our understanding in this topic. Only when there is adequate knowledge regarding the fundamental pathophysiology behind AMI built from benchwork, can we form clinically relevant questions to better evaluate the use of antibiotics in real-life scenario. To further diverge, the question is not simply IF antibiotics use is beneficial in AMI patients, but WHY and HOW, if the answer to first question is yes. For example, a future study could include subdivision of the patients by extent or anatomic region of ischemia to potentially elucidate if antibiotic driven management potentially abrogates the need for exploratory laparotomy in cases of AMI affecting certain regions of the viscera versus others. In précis, more work needs to be done, and we hope this review of the literature as well as scientific motivations for antibiotics usage in AMI serves as a call to action.

**Table 1:** Summary of Selected Clinical Literature on AMI and Antibiotics

Study Name	Study Publish Year	Study Inclusion Period	Study Type	Institutions	Patients	Antibiotic Usage	Study Findings or Commentary Regarding Antibiotics
Acute superior mesenteric ischaemia [47]	1987	1973–1984	Retrospective	Single Institution	102	Not mentioned.	None
Failure to Improve Outcome in Acute Mesenteric Ischaemia: Seven Year Review [48]	1999	1987–1993	Retrospective	Single Institution	57	Not mentioned.	None
Surgical Management of Thrombotic Acute Intestinal Ischemia [49]	2001	1993–2000	Retrospective	Single Institution	170	Not mentioned.	Endorses prophylactic antibiotic usage in Discussion.
Endovascular and open surgery for acute occlusion of the superior mesenteric artery [50]	2010	1999–2016	Retrospective	Multicenter (28 hospitals)—Swedevasc Database	163	Not mentioned.	None
A comparison of endovascular revascularization with traditional therapy for the treatment of acute mesenteric ischemia [13]	2011	1999–2008	Retrospective	Single Institution	70	Not mentioned.	None
A study on 107 patients with acute mesenteric ischemia over 30 years [51]	2012	1980–2010	Retrospective	Single Institution	107	Not mentioned.	Endorses prophylactic antibiotic usage in Discussion.
Effects of a Multimodal Management Strategy for Acute Mesenteric Ischemia on Survival and Intestinal Failure [41]	2013	2009–2011	Prospective	Single Institution	18	All patients received oral digestive decontamination with PO gentamicin and metronidazole. Patients with SIRS received IV piperacillin-tazobactam.	Notes that 11 patients did not require intestinal resection, and postulates that this maybe due to “prompt pathophysiological-based medical protocol”.
Risk Factors Effecting Mortality in Acute Mesenteric Ischemia and Mortality Rates: A Single Center Experience [52]	2013	2006–2011	Retrospective	Single Institution	95	All patients received prophylactic antibiotics.	None
Comparison of open and endovascular treatment of acute mesenteric ischemia [53]	2014	2005–2009	Retrospective	Multicenter (~ 1000 hospitals)—National Inpatient Sample Database	4,665	Not mentioned.	None
Endovascular Therapy for Acute Mesenteric Ischemia: an NSQIP Analysis [15]	2015	2005–2010	Retrospective	Multicenter (~ 300 hospitals)—National Surgical Quality Improvement Program Database	3,689	Not mentioned.	None
The importance of open emergency surgery in the treatment of acute mesenteric ischemia [54]	2015	2001–2014	Retrospective	Single Institution	54	Not mentioned.	None
Prognostic factors in patients with acute mesenteric ischemia [55]	2017	2014	Retrospective	Single Institution	46	Not mentioned.	Endorses prophylactic antibiotic usage in Discussion.
Primary Endovascular Intervention for Acute Mesenteric Ischemia Performed by Interventional Cardiologists – A Single Center Experience [56]	2017	2012–2014	Retrospective	Single Institution	8	Broad spectrum antibiotics were given at the discretion of critical care specialist after stenting. No criteria given for their usage.	None
Endovascular Treatment for Acute Thromboembolic Occlusion of the Superior Mesenteric Artery and the Outcome Comparison between Endovascular and Open Surgical Treatments: A Retrospective Study [17]	2017	2007–2012	Retrospective	Single Institution	30	Not mentioned.	None
Oral Antibiotics Reduce Intestinal Necrosis in Acute Mesenteric Ischemia: A Prospective Cohort Study [27]	2019	2009–2015	Prospective	Single Institution	67	PO or IV antibiotics were given at the discretion of the admitting physician.	Oral antibiotics in addition to early revascularization can reduce progression of AMI to intestinal necrosis.
Prognostic factors of acute mesenteric ischemia in ICU patients [46]	2019	2000–2017	Retrospective	Single Institution	214	Stated that 90% of patients received antibiotic therapy.	Reported that receiving antibiotic therapy was not significantly associated with survival.
Challenges Encountered during the Treatment of Acute Mesenteric Ischemia [11]	2020	2002–2018	Retrospective	Single Institution	43	Mentions 34 patients requiring IV antibiotic therapy in results. Does not detail criteria for treatment.	Notes that patients requiring antibiotic therapy were at significantly increased risk of death.
Risk factors of geriatrics index of comorbidity and MDCT findings for predicting mortality in patients with acute mesenteric ischemia due to superior mesenteric artery thromboembolism [57]	2020	2013–2018	Retrospective	Single Institution	33	Not mentioned.	None
Interdisciplinary approach in emergency revascularization and treatment for acute mesenteric ischemia [44]	2021	2010–2017	Prospective	Single Institution	26	Not mentioned.	None
The implementation of a pathway and care bundle for the management of acute occlusive arterial mesenteric ischemia reduced mortality [42]	2021	2014–2020	Retrospective	Single Institution	145	All patients treated after implementation of clinical pathway received broad-spectrum antibiotics. No information regarding pre-pathway treatment.	None
A Shifting Trend Towards Endovascular Intervention in the Treatment of Acute Mesenteric Ischemia [58]	2021	2007–2018	Retrospective	Single Institution	98	Not mentioned.	None

## Data Availability

Not applicable.
